# Supplementary material for: A Newly Engineered A549 Cell Line Expressing ACE2 and TMPRSS2 Is Highly Permissive to SARS-CoV-2, Including the Delta and Omicron Variants
Source: Viruses. 2022 Jun 23;14(7):1369. doi: 10.3390/v14071369 (PMC9318744; doi:10.3390/v14071369)
Supplement: Supplementary file 1 [file viruses-14-01369-s001.zip › viruses-1760272-supplementary.pdf]

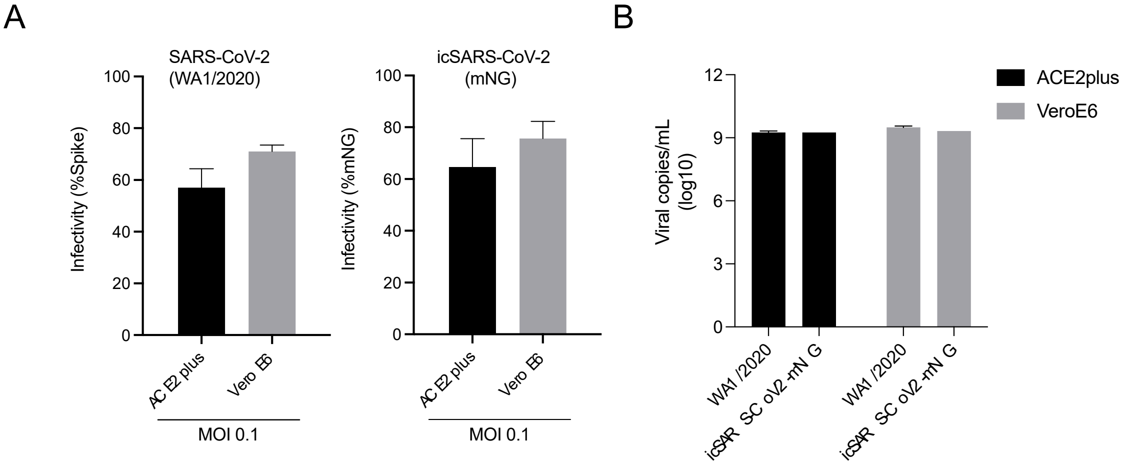

Figure S1. ACE2plus cells show comparable infectivity to Vero E6 cells**.** (A) Cells were challenged with SARS-CoV-2 (WA1/2020) or icSARS-CoV-2-mNG (mNG) for 48 hours, then the percentage of infected cells was quantified by ImageXpress to measure the infectivity. (B) Virus-containing supernatants from infected ACE2plus and VeroE6 cells were collected and analyzed by RT-qPCR to determine the viral copy number. The data represent the mean ± SD.

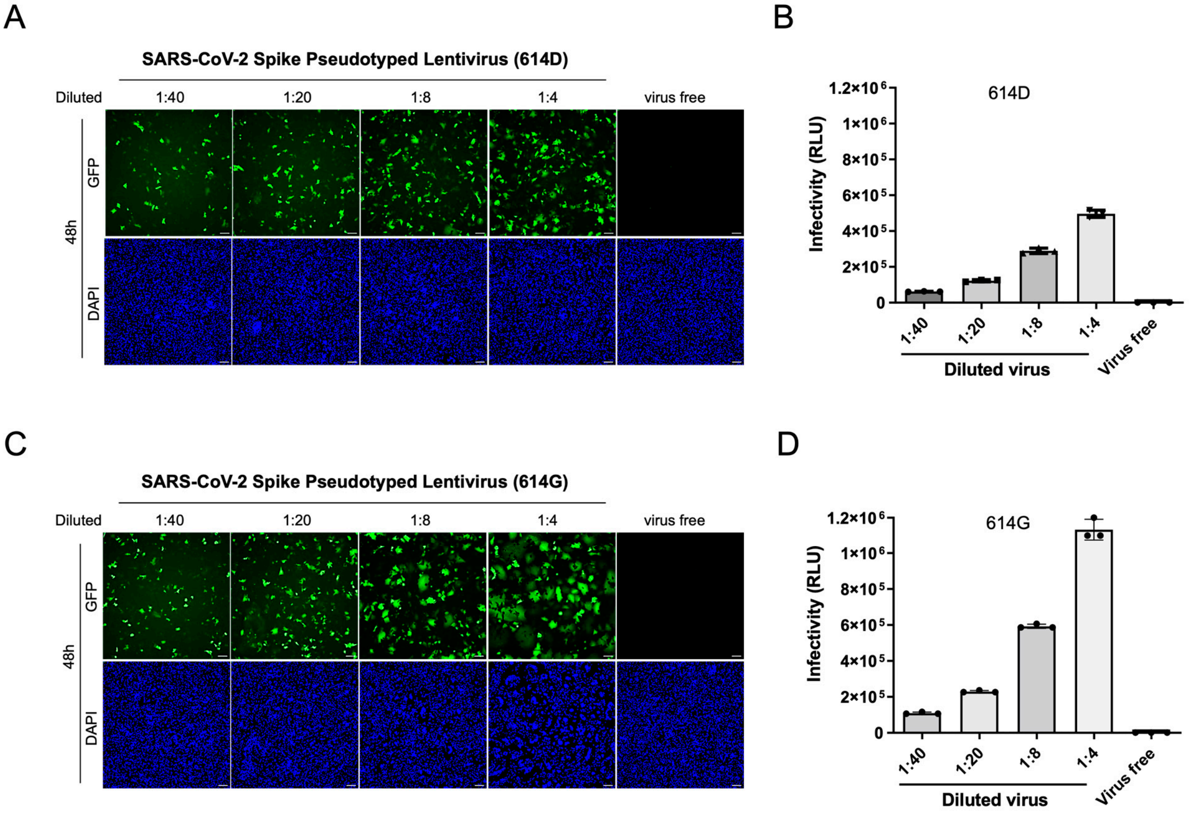

Figure S2. ACE2plusC3 cells can be infected with SARS-CoV-2 Spike-pseudotyped lentivirus (PV). Representative images of ZsGreen expression in ACE2plusC3 cells at 48 hours post-incubation with a series of diluted 614D or 614G PV are shown (A, C). Infectivity was measured via relative luciferase units (RLU) as shown in B and D. Each data represents the mean and standard deviation. 100 µM scale bar.

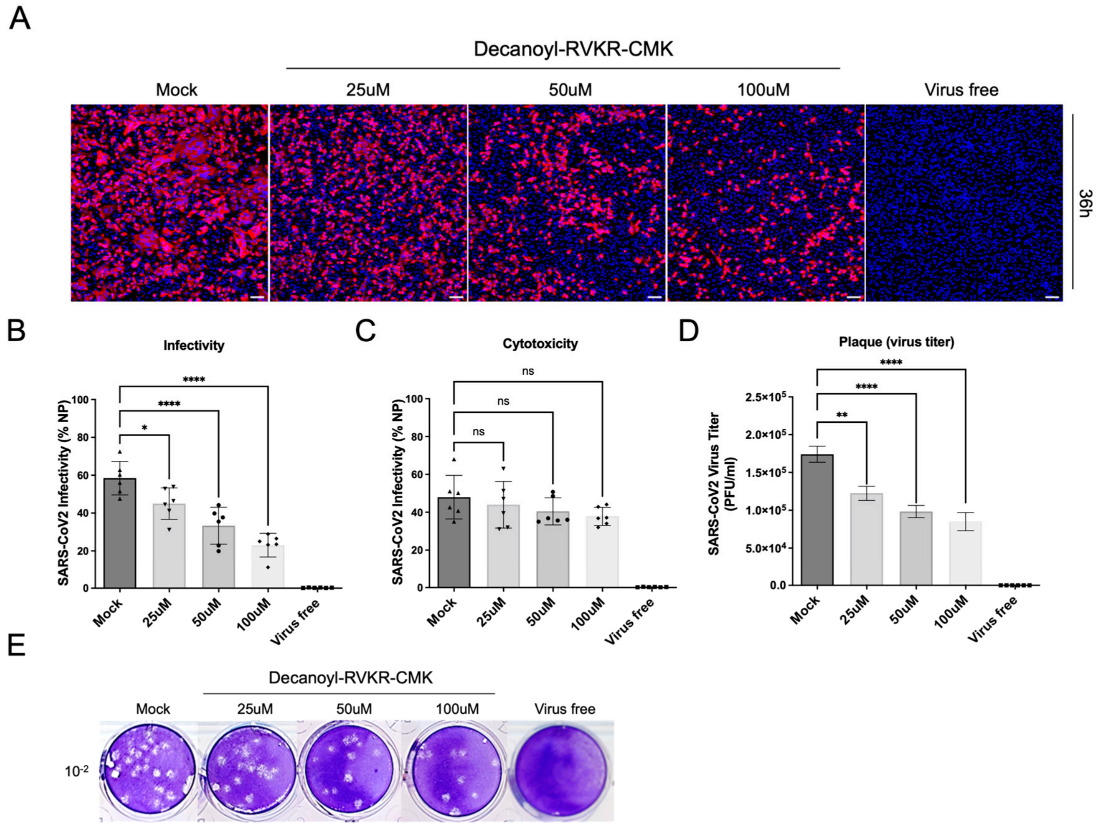

Figure S3. The furin inhibitor decanoyl-RVKR-CMK moderately inhibits SARS-CoV-2 infection in ACE2plusC3 cells. (A) Microscope images show viral nucleocapsid protein (NP) expression (red) in infected cells, with DAPI (blue) used for nuclear counterstaining. Cells were infected with SARS-CoV-2 (WA1/2020) at an MOI of 0.1 in the presence of furin inhibitor for 36 hours. Images were scanned by ImageXpress using 10x magnification. (B) Quantifying the virus infectivity via ImageXpress. (C) Cytotoxicity was measured by the LDH assay. (D, E) Viral titer quantification by plaque assay of supernatants collected at 36 hours post-infection and example images are shown. Data are representative of the mean and SEM of two independent experiments. ns, not significant; *, $p < 0.05$; **, $p < 0.01$; ****, $p < 0.0001$.
